# Decrement of Transcriptome Level in Epithelial Tight Junction Claudin and Occludin as an Epithelial-Mesenchymal Transition Signature for Colorectal Cancer Biomarker: Colorectal Cancer Diagnostic Markers

**DOI:** 10.31661/gmj.v11i.2350

**Published:** 2022-12-31

**Authors:** Maryam Ghoojaei, Reza Shirkoohi, Mojtaba Saffari, Amirnader Emamirazavi, Mehrdad Hashemi

**Affiliations:** ^1^ Department of Biology, University of Central Florida, Florida, United States; ^2^ Cancer Biology Research Center, Cancer Research Institute, Imam Khomeini Hospital Complex, Tehran University of Medical Sciences, Tehran, Iran; ^3^ Department of Genetics, Faculty of Medicine, Tehran University of Medical Sciences, Tehran, Iran; ^4^ Iran National Tumor Bank, Cancer Institute of Iran, Tehran University of Medical Sciences, Tehran, Iran; ^5^ Department of Genetics, Tehran Medical Sciences, Islamic Azad University, Tehran, Iran; ^6^ Farhikhtegan Medical Convergence Sciences Research Center, Farhikhtegan Hospital Tehran Medical sciences, Islamic Azad University, Tehran, Iran

**Keywords:** Colorectal Cancer, Metastasis, Gene Expression, Claudins, Occludin

## Abstract

**Background:**

Colorectal cancer is a common and fatal disease worldwide with increasing diagnosed cases yearly. Moreover, about 90% of deaths associated with cancers occur due to metastasis, which overcomes tight junction proteins such as claudin and occludin. The present study aimed to evaluate the significance of claudin and occludin expression change in human colorectal cancer.

**Materials and Methods:**

In this case-control study, 38 colorectal cancer patients were compared with normal samples regarding the expression levels of claudin and occludin genes by polymerase chain reaction.

**Results:**

The expression levels of claudin and occludin significantly decreased in tumor samples compared to normal samples.

**Conclusion:**

The change in the expression level of the claudin and occludin genes could be considered an influential factor in turning normal healthy tissues into cancerous cells.

## Introduction

Approximately two million new cases of colorectal cancer (CRC) are diagnosed every year, making it the third most common cancer and the fourth most common cause of cancer-related death, with 700,000 deaths annually [1][2]. Since there is mostly no distinct symptom, and the lower abdominal signs are common and often associated with non-neoplastic conditions, CRC diagnosis is challenging in all health systems. Colonoscopy is a common method of CRC diagnosis, which is an invasive procedure [3]. Following the cancer diagnosis, the staging process determines whether the CRC has remained within the intestine or has spread to other parts of the body [4]. Metastasis is a complex process involving the dissociation of tumor cells from the primary tumor, invasion, migration, entrance into blood vessels, survival in the blood vessels, entrance to the parenchymal tissue of the target organ, and the formation of a colony in the secondary location [5]. The epithelial-mesenchymal transition (EMT) is a process happening during development, fibrosis, and wound healing. It can also play a role in preserving cancer and inducing metastasis by changing the cell-to-cell connection and cytoskeleton in the extracellular matrix and releasing epithelial cells from the peripheral tissue [6][7]. Transcription factors facilitate EMT by repressing the promoters of the tight junction proteins [8][9][10]. Claudins, along with occludin, are the most important proteins in the tight junction (zonulae occludent) structure [11][12], and their abnormal expression is an indicator of increased invasion and reduced cell adhesion [13][14]. For instance, loss of occludin was confirmed to lead to the progression of human breast cancer [15]. In another study, occludin expression was analyzed in human colorectal liver metastasis, and significant downregulation of occludin expression was seen in tumoral samples [16]. Moreover, the increased expression of claudin-1, -3, and -4 are associated with tumor depth in the CRC tissues [17][18]. Also, Mandle et al. evaluated the impacts of calcium and vitamin D on tight junction proteins of the CRC patients under treatment and found increased expression of claudin-1 and occludin [19].

Considering the increasing frequency of CRC and metastasis-which causes about 90% of cancer deaths-early detection of CRC is helpful to offer the appropriate treatment interventions and consequently control the disease [20]. In addition, since the current standard CRC diagnostic methods are invasive, identifying biomarkers indicating invasive cells is important for prophylaxis and treatment. The present study aimed to assess the changes in the expression of claudin and occludin in different stages of CRC samples to identify diagnostic biomarkers and address the correlation of the EMT phenomenon with metastasis.

## Materials and Methods

**Table:** Table1. Primers Used for Polymerase Chain Reaction

**Genes**	**Sequences (5´-3´)**	**Primer length (kb)**	**Product length (kb)**
***Claudin***	F: TCGATACAATGGCACAGTGG	20	182
	R: CAATCCCGCTATTGTGGTTT		
***Occludin***	F: TCCAATGGCAAAGTGAATGA	20	182
	R: GCAGGTGCTCTTTTTGAAGG		
**GAPDH**	F: TCACCAGGGCTGCTTTTAAC	20	152
	R: GACAAGCTTCCCGTTCTCAG		

**GAPDH:** Glyceraldehyde-3-phosphate dehydrogenase

### Tissue Samples Collections

The present case-control study was performed on patients who were referred to the Cancer Institute of Imam Khomeini Hospital, Tehran, Iran. Briefly, 38 tissue specimens from patients with CRC and three normal samples were randomly selected from the National Tumor Bank of Iran, affiliated with the Cancer Research Institute. The normal samples were from adjacent tissues far from the margin of tumor tissues from the same patients and were collected as controls to compare with tumor samples regarding gene expression. The collected tissue samples were from different colon regions, including the rectosigmoid, rectum, sigmoid colon, descending colon, transverse colon, ascending colon, and cecum. A pathologist evaluated all the histological information of the samples, including the stage of disease, grade, size, and metastasis. Hence, the TNM staging system (T indicates tumor size, N expresses lymph node involvement, and M means metastasis) was applied to assess the cancer stage. Tissue samples were stored at -80 °C after dissection to prevent RNA degradation.

### RNA Extraction, cDNA Synthesis, and Quantitative Real-Time Polymerase Chain Reaction (PCR)

The RNA was extracted using an easy-BLUE RNA extraction kit (iNtRON Biotechnology, Seoul, Korea). The optical density and absorption of RNA were measured at a wavelength of 260 and 280 nm by a nanodrop machine (Spectrophotometer, 2000, Thermo Fisher Scientific Inc., Wilmington, USA). Samples with a 260/280 ratio equal to or greater than 1.7 were chosen to synthesize the complementary DNA. On agarose gel electrophoresis, RNA revealed its good quality. Then, the cDNA was prepared using the Random Hexamer primer. Table-1 shows the PCR primers designed by Primer3 software [21] for claudin and occludin as targets and glyceraldehyde-3-phosphate dehydrogenase (GAPDH) as the housekeeping gene for qPCR. Real-time PCR (Rotor-Gene Q real-time PCR cycler, QIAGEN Inc., USA) was performed (initial denaturation in 95 °C for 15 minutes, and denaturation in 95 °C for 15 seconds, annealing for 1 minute for claudin and occludin in 55 °C and 60 °C, respectively, and elongation in 72 °C for 20 seconds in 40 cycles) to get the Ct (cycle threshold) values to estimate the fold change in expression of the desired genes [22].

### Ethical Considerations

All procedures performed in this study were in accordance with the ethical standards of the Cancer Research Institute, Tehran University of Medical Sciences (ethical approval: IR.TUMS.IKHC.REC.1401.018), and informed consent was obtained from all patients.

### Statistical Analysis

To evaluate any changes in the gene expression and to make comparisons, the relative expression software tool (REST, QIAGENE Inc., USA) was used [23]. This method helps with standardizing the expression of target genes. Finally, the relative gene expression data analysis was performed using the comparative Ct(2-ΔΔCT) method [24]. Also, the t-test was performed using RStudio 1.2.1335 [25] to evaluate the difference between the mean expression of claudin and occludin and the disease stage, tumor grade, tumor size, and metastasis. A P-value≤0.05 was considered as significant difference.

## Results

**Figure-1:**
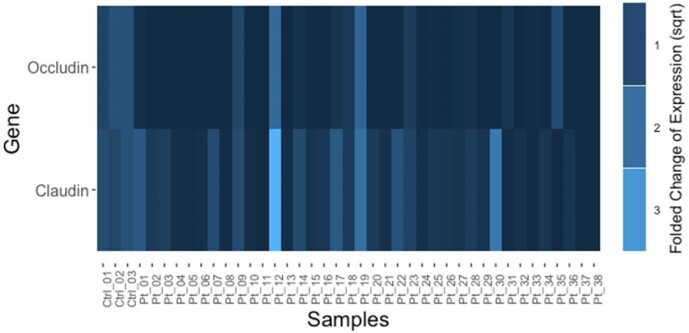
Figure 1. The folded change of claudin and occludin expression (sqrt) across all the samples

**Figure-2:**
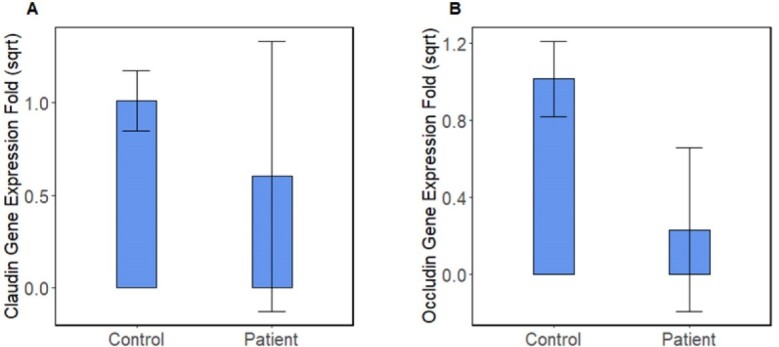
Figure 2. The mean gene expression for claudin (A) and occludin (B) in studied samples

**Figure-3:**
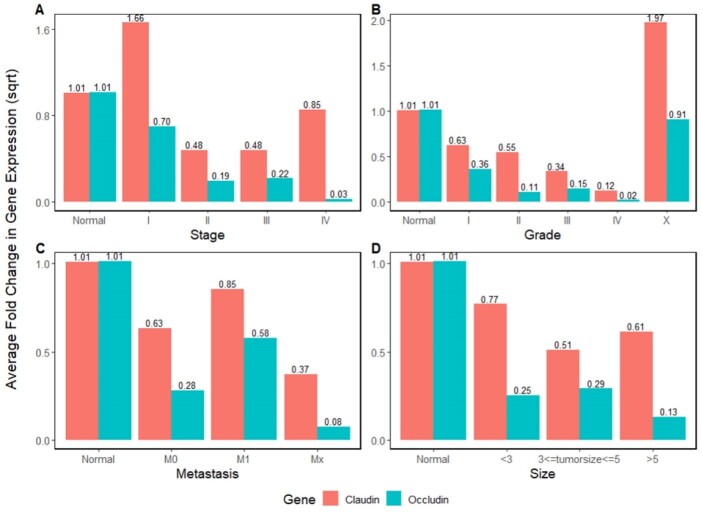
Figure 3. The mean fold change in claudin and occludin expressions across different stages (A), grades (B), metastasis state (C), and tumor size (D) compared to normal samples

**Table:** Table2. Patients Histopathological Characteristics

**Variables**		**Number**	**Percent**
**Age, y**	<40	6	15.78
	≥40	32	84.22
	<3	8	21.05
**Tumor size (cm)**	3≤size≤5	16	42.1
	>5	14	36.85
	I	11	28.94
	II	18	47.39
**Grade**	III	6	15.78
	IV	1	2.63
	X	2	5.26
	I	3	7.89
**Stage**	II	16	42.1
	III	16	42.1
	IV	3	7.91
	M0	29	76.31
**Metastasis**	Mx	6	15.78
	M1	3	7.91

**Mx:** Properly metastasis

### Histopathological Characteristics of Patients

Histopathological characteristics of patients were received from the National Tumor Bank of Iran. The mean age of patients was 55 years, with the maximum and minimum ages of 79 and 16 years, respectively. The patient clinical characteristics are presented in Table-2.

### Claudin and Occludin Expression

We used the Ct values of GAPDH and each claudin and occludin gene to estimate the fold change in gene expression using 2-ΔΔCT. Then, we transformed the data using square root to have homogeneous data with normal distribution. Figure-1 shows the fold change of gene expression for claudin and occludin in normal (control) and CRC samples. We observed that most of the samples had lower occludin expression than normal tissues, while the majority of the samples had claudin expression similar to normal tissues.

As shown in Figure-2A, the mean claudin expression in CRC was lower than in the control group (0.6±0.73 vs. 1.01±0.16). Also, according to Figure-2B, the mean occludin expression in the patients group (0.23±0.42) was lower than in the control group (1.01±0.2). The t-test indicated that claudin and occludin expression were significantly different between the control and patients groups (P=0.02 and P=0.005, respectively).

Regarding claudin, the mean gene expression in normal tissues was 1.01±0.16. Although the mean claudin expression was increased in stage I (1.66±1.74, Figure-3A), it decreased in stages II (0.48±0.43), III (0.48±0.52), and IV (0.85±1.19). Regarding occludin, the mean expression was decreased in stages I (0.70±0.97), II (0.19±0.29), III (0.22±0.43), and IV (0.02±0.009) compared to the normal samples (1.01±0.2, Figure-3A). There was a significant difference in claudin expression level between normal samples and stages II and III (P=0.005 and 0.007, respectively). Moreover, occludin expression was significantly different between the normal samples and stages II, III, and IV (P=0.004, P=0.002, and P=0.013, respectively).

As mentioned in Figure-3B, mean claudin expression decreased in grades I (0.63±0.53), II (0.55±0.63), III (0.33±0.21), and IV (0.12), while it increased in grade X (1.97±2.36) compared to normal samples (1.01±0.16). Also, the occludin expression decreased in grades I (0.36±0.51), II (0.11±0.22), III (0.15±0.19), IV (0.02), and X (0.9±1.28) compared to normal samples (1.01±0.2, Figure-3B). The t-test confirmed the statistically significant difference in claudin expression between the normal samples and grades II and III (P=0.02 and P=0.003, respectively) and occludin expression between the normal samples and grades I, II, and III (P=0.007, P=0.006, and P=0.003, respectively).

Figure-3C represents the change in mean gene expression in different metastasis states. Data showed that claudin expression for normal samples was 1.01±0.16, while it significantly decreased for non-metastatic (0.63±0.73), metastatic (0.85±1.19), and tissues with metastasis probability (0.37±0.55). In addition, occludin expression decreased in all metastasis states of M0 (0.28±0.47), Mx (0.08±0.1), and M1 (0.58±1.1) compared to normal samples (1.01±0.19, Figure-3C).

Figure-3D shows the mean gene expression change across three tumor size categories. Regarding claudin, the mean expression decreased in tumors smaller than 3 cm (0.77±1.22), between 3 to 5 cm (0.51±0.53), and greater than 5 cm (0.61±0.61) compared with normal tissue (P=0.009). Also, occludin expressions were decreased in all three tumor size categories compared to normal samples (Figure-3D).

## Discussion

We compared the fold change in gene expression between normal and CRC tumor tissues for claudin and occludin, which are the most critical proteins in tight junctions [26]. Although we had a limitation in control sample size due to the limited available normal mucosae far from the margin of tumor tissues in the tumor bank, our results indicated that the mean expression of both claudin and occludin decreased in CRC samples compared to normal tissues. Regarding the stage of the disease, we observed significant differences for stages II and III compared to normal samples, which can be attributed to the lower number of samples in other stages. The changes in claudin and occludin expression levels may have prognostic significance in patients with CRC, as it was identified in other cancers. Ganjzadeh et al. revealed that an increase in the expression level of the occludin gene in breast cancer tissues was considered to one of the effective factors in tissue transformation toward the cancerous phase [27].

Moreover, Park et al. suggested that occludin expression was highly related to the development of peritumoral brain edema and can consider as a prognostic factor [28]. Salehi et al. showed that the downregulation of occludin expression in patients with melanoma was an authentication mark of cancer progression [29]. In addition, Phattarataratip et al. indicated that claudin-7 expression could effectively predict the prognosis of oral squamous cell carcinoma [30]. Furthermore, it was proposed that claudin-1 is probably a molecular marker in squamous cervical cancer and potentially can be a diagnostic, prognostic, and therapeutic marker [31]. Also, higher expression of claudin-3 was observed among patients with metastasis of prostate cancer [32].

In the current study, we showed significant differences in occludin expression in all available tumor sizes compared to normal samples; however, significant differences in claudin expression were seen only in the tumor size 3 to 5 cm. Indeed, the expression level change with increased tumor size can indicate CRC progress. Evidence suggested that the protein expression of claudin 1, 3, 4, 5, and 7 could associated with tumor growth patterns in colon carcinoma; hence, it may have a progressive effect on colon carcinoma development and can consider a tumor marker [33]. Niknami et al. mentioned that vimentin expression decreased in CRC with larger tumor sizes, and increased expression of fibronectin was correlated with high tumor stages [34].

The significant decrease in occludin expression in samples with metastasis can indicate that with loss of occludin, the metastasis can be facilitated as a result of a weakened tight junction [35]. In the current study, the number of metastatic specimens was insufficient to make a reasonable conclusion; however, as shown previously, claudin protein was highly expressed in liver metastases of colorectal adenocarcinoma [36].

## Conclusion

Based on the results of this study, a significant change in the expression level of claudin and occludin in CRC specimens compared to normal samples was specified, which might be useful and reliable for the detection of CRC and its probable prognosis.

## Conflict of Interest

The authors have no conflict of interest in this work.
